# Adolescents’ Experience during Brace Treatment for Scoliosis: A Qualitative Study

**DOI:** 10.3390/ijerph191710585

**Published:** 2022-08-25

**Authors:** Mei-Chun Cheung, Derry Law, Joanne Yip, Jason Pui Yin Cheung

**Affiliations:** 1 Department of Social Work, The Chinese University of Hong Kong, Shatin, New Territories, Hong Kong SAR, China; 2 Department of Design, Caritas Institute of Higher Education and Caritas Bianchi College of Careers, Tseung Kwan O, New Territories, Hong Kong SAR, China; 3 School of Fashion and Textiles, The Hong Kong Polytechnic University, Hung Hom, Kowloon, Hong Kong SAR, China; 4 Department of Orthopaedics and Traumatology, The University of Hong Kong, Hong Kong SAR, China

**Keywords:** adolescent idiopathic scoliosis, brace treatment, subjective experiences

## Abstract

This study aimed to explore the subjective experiences of adolescents with scoliosis during brace treatment in order to understand their obstacles and make recommendations to enhance brace compliance. Using purposive sampling, 15 adolescents (2 males and 13 females) with scoliosis aged from 10 to 16 years old during brace treatment were recruited to participate in semi-structured in-depth interviews. The data were recorded, transcribed, and coded using thematic analysis with the qualitative software NVivo 10. Significant statements and phrases were organized into categories and themes to understand adolescents’ experiences during brace treatment for scoliosis. In general, the adolescents acknowledged that compliance with brace treatment was essential to reduce or prevent the progression of spinal curvature and tried their best to comply with the treatment. Regarding their subjective experiences during brace treatment, three themes were identified and emerged as obstacles negatively affecting their brace compliance, including physical discomfort due to brace materials and design, reluctance caused by the brace’s visual appearance, and passive patient participation during the treatment process. This study reveals insights into the experiences of adolescents with scoliosis during brace treatment and what they perceive as hindrances to compliance. In order to have better brace compliance, adolescents’ feelings and difficulties during brace treatment should be recognized and addressed. Therefore, active patient participation throughout the treatment process, involving the co-design of a customized brace, psychosocial interventions, and personalized appearance style management should be considered and promoted to facilitate a more acceptable bracing experience to achieve better brace compliance.

## 1. Introduction

Adolescent idiopathic scoliosis (AIS) is characterized by curvature of the spine and asymmetry in the shoulders and hips. The basic management of AIS consists of surgical treatment, orthotic spinal bracing, or clinical monitoring [1]. Since severe spinal deformities can considerably reduce pulmonary and cardiac functions, surgical treatment is generally recommended when the Cobb angle of the spine is greater than 45–50° [2]. The Cobb angle is the gold standard to assess the severity of the spinal deformity and is measured by the angle between the two most-tilted spinal vertebrae in the spinal curve. Non-surgical treatment, such as immobilization with a spinal brace, remains the most effective treatment modality to prevent the progression of spinal curvature for adolescents with a Cobb angle between 20° and 40° [3]. However, to effectively control the progression of spinal curvature, the brace should be worn for up to 23 h a day until the adolescent stops growing, which means that the treatment may last for 4–6 years [4].

Bracing prevents the need for surgery for most AIS patients, but compliance is the main obstacle to success. The brace’s rigidity and prolonged wear duration have significantly impeded patients’ compliance during the treatment period. Although more than 90% of adolescents indicated that their main motivations for compliance were the desire to avoid surgery and to prevent curve progression, around 40% of adolescents, as reported in a questionnaire, complained of physical discomfort, including back pain while wearing the brace and pain from the brace rubbing/causing pressure points [5]. Brace-related stress is expressed by adolescents with scoliosis, mainly due to the compression and impairment of breathing felt by adolescents [6]. Adolescents experience a medium stress level due to the brace rather than the deformity [7]. In addition, bracing has adverse effects on the psychosocial well-being of adolescents with scoliosis [8], involving the worsening of their body image [9,10] and a reduction in their quality of life [9,10,11]. Sapountzi-Krepia et al. [12] found that adolescents with scoliosis who received brace treatment had poorer perceptions of their body image and lower levels of happiness and satisfaction than a control group. The adverse effects on the psychosocial well-being of adolescents with scoliosis caused by brace treatment result in poor compliance with bracing [13,14]. However, a multicenter, randomized controlled clinical trial, namely the Bracing in Adolescent Idiopathic Scoliosis Trial [15], did not support findings from previous research suggesting that bracing has significant negative impacts on psychosocial well-being, including body image and quality of life.

To achieve better treatment outcomes, patient participation and involvement in the treatment are crucial, as it has been found to have a positive effect on the process and rehabilitation outcomes [16]. Active participation and shared decision-making in healthcare can potentially increase adolescents’ engagement and compliance with treatment and enable them to play a more significant role in the treatment process [17]. In fact, our previous study on adolescents with bracing, referred by a certified prosthetist/orthotist for interview between 2015 and 2016 [18], demonstrated that co-designing a brace with adolescents concerning the aesthetic aspects of the surface design of the brace induced positive subjective experiences in adolescents, addressing their psychological issues during treatment and increasing user compliance. While most previous studies on the motivation for compliance with bracing in adolescents with scoliosis have focused on the impact of a brace on adolescents’ physical and psychological functions [5,6,8], studies on adolescents’ views of their involvement in the treatment process are relatively limited. Therefore, this study addressed this research gap by conducting a qualitative study to explore the subjective experiences of adolescents during brace treatment for scoliosis to understand their obstacles to compliance and their perspectives on patient participation in the treatment process.

## 2. Materials and Methods

### 2.1. Participants

A total of 15 adolescents (2 males and 13 females) were recruited from one of two referral centers in the territory-wide scoliosis screening program since 2018. It is a specialized center for the treatment of paediatric orthopaedic and spine deformity problems for children/adolescents. Adolescents undergoing brace treatment were invited to participate in the study voluntarily during their medical follow-up consultations at the Department of Prosthetic and Orthotic of the specialized center by one of the authors (D.L.), who had no prior interaction with any adolescent. All adolescents were under treatment with custom-molded semi-rigid thoracolumbosacral orthosis. The brace was prefabricated based on the adolescent’s curve shape and flexibility with lumbar and thoracic pressure pads. Informed consent from the adolescents and informed written consent from their parents for participation were solicited before the interviews. The demographic and clinical information of the adolescents is shown in Table 1. The mean age of the adolescents at the interview was 13.0 years (SD = 1.59), and the average Cobb angle was 33.9° (SD = 5.06). The mean duration of bracing was 13.33 months (SD = 9.17). The study protocol was approved by the Human Subjects Ethics Sub-committee of The Hong Kong Polytechnic University, the Survey and Behavioural Research Ethics Committee, and the Joint Chinese University of Hong Kong–New Territories East Cluster Clinical Research Ethics Committee of The Chinese University of Hong Kong.

### 2.2. In-Depth Interview

A qualitative approach was chosen to understand the subjective experiences of adolescents with scoliosis during brace treatment. All interviews were conducted face-to-face individually in Cantonese. The adolescents attended the in-depth interviews in the presence of a parent. The interviews took place at prearranged dates and times in an interview room in the specialized center and lasted approximately 55–75 min. A semi-structured interview design and probing were applied to understand and clarify the adolescents’ subjective experiences during brace treatment. The interview mainly covered three domains, including (1) clinical information about scoliosis, (2) adolescents’ views on the obstacles towards brace compliance, and (3) their subjective experiences during brace treatment. The verbatims were recorded and transcribed into Chinese.

### 2.3. Data Analysis

The verbatims were analyzed by thematic analysis with the qualitative software NVivo 10. We chose to conduct a thematic analysis because it can identify patterns across the data from life experiences and help us understand how users feel and think [19]. This method is highly inductive [20]. The meaningful and essential statements of the interview data were extracted, coded, categorized, and arranged into themes by two authors (M.-C.C. and D.L.) to identify patterns and interpretations of meanings across the data to understand participants’ subjective experiences about the treatment process. The themes and descriptions were then reviewed by other authors (J.Y. and J.P.Y.C.) to ensure that detailed descriptions were included. The sample size was determined by data saturation when no further information relating to the themes emerged. Having interviewed 15 adolescents with scoliosis who had undergone brace treatment, the data collection stopped as repetitive information from the adolescents was identified.

## 3. Results

The thematic analysis generated three themes to illustrate the obstacles that adolescents faced regarding brace compliance based on their subjective experiences: (1) physical discomfort due to brace material and design, (2) reluctance caused by the brace’s visual appearance, and (3) passive patient participation during the treatment process. The quotes reported in this section were translated from the Chinese transcriptions into English.

### 3.1. Theme 1: Physical Discomfort Due to Brace Material and Design

The adolescents stated that the hardness and low breathability of the material used for braces resulted in physical discomfort. In fact, the physical design of the brace made them feel so uncomfortable and caused so much pain that they were unwilling to wear it for an extended period. Specifically, the hardness of the bracing material and the tight-fitting design of the brace restricted upper trunk movement in their daily life.

*“It feels annoying to wear the brace as it is stifling, especially in summer. I may take it off in the physical education lesson, but it depends on the teacher. If the teacher is female, she tends to understand the situation (bracing). If the teacher is male, he does not seem to understand my situation.”*

(A1)

*“I was quite worried about the comfort of wearing it (brace) since it looked stiff from the first impression. I knew I had to wear it for twenty hours daily, and I felt that I might be unable to move around after wearing it.”*

(A5)

*“The material is so hard that makes me painful, and it would be better if softer material can be used.”*

(A8)

In addition, since Velcro is a crucial design feature for controlling the opening and closing of the brace, affecting the corrective function of the brace, adolescents complained about the durability of the Velcro. They felt very irritated and frustrated when it became worn out after being used for a while. As a result, the adolescents had to return to the specialized center frequently for repairs and/or replacements.

*“The Velcro backstrap is easy to wear out, and the material is not durable after using it for around three months. It can’t tighten up the brace. When I take a deep breath, it will fall apart. I have to come back (to the hospital) for a few times to replace the straps.”*

(A11)

### 3.2. Theme 2: Reluctance Caused by the Brace’s Visual Appearance

Before the treatment process started, the psychological preparation of the adolescents toward brace treatment was found to be important. Most adolescents did not understand the significance of bracing clearly and thoroughly or how a brace looked at the beginning of the treatment process. When they were informed about their spinal deformity and saw the rigid brace placed in front of them, most of the adolescents expressed that they were shocked and scared of the visual appearance of the brace, triggering their reluctance to wear it.

*“I cried when I saw the brace first time. The appearance made me so unhappy that I thought it would be seen by others when wearing it throughout the treatment. It made me so uncomfortable when I saw the brace.”*

(A6)

Among the visual elements, the color of the brace created the most substantial negative impact on the emotional responses of the adolescents toward the brace, further intensifying their anxiety and stress as they perceived the brace as a prosthetic.

*“The flesh-colored brace makes me feel so bad.”*

(A1)

*“I felt shocked seeing the brace first time. The color makes me feel like I am a patient who needs to wear prosthetics.”*

(A4)

*“The color of the brace is strange, it is not like the skin tone of human beings, it is artificial and odd. I don’t want to wear it.”*

(A5)

*“Though the brace is in nude color, the tone does not match my skin tone, and it would be seen by others.”*

(A12)

Moreover, the adolescents were concerned about their self-image and body configuration and were always worried about whether others would see the brace. They felt embarrassed and upset when the brace could not be hidden. As a result, most of them preferred to wear dark colors and loose-fitting clothes to conceal the brace, though these styles and colors were not their preference or fitted to their bodies. One participant expressed that the brace looked ugly, so she felt shy and anxious if it could be seen by others, especially when she wore a sheer summer school uniform.

*“My intention to wear more clothes to hide the brace is higher when the discomfort happened. I don’t want my friends to see it.”*

(A9)

*“I like wearing dark-colored clothing now to hide the brace. If wearing pale colors, others will see my brace. Especially in summer, the material of the clothing is sheer, and only black color can hide it.”; “I also wear high-necked top and drawstring pants, etc.”*

(A12)

*“I wear a sweater to school even in summer to hide the brace since my school uniform is in white, my classmates will see it.”*

(A13)

*“I like pink and girly clothing styles, but my mum would choose loose-fit and black clothing styles so that my brace will be hidden… If the color is white that will match with my school uniform, and it can be hidden.”*

(A14)

Though male adolescents did not demonstrate strong reluctance towards the color of the brace, its visual appearance did affect their initial impression of bracing. While the reluctance was quite long-lasting for female adolescents, male adolescents demonstrated psychological adaptation towards the brace after a while.

*“My first impression on the brace was weird and heaving from its appearance, and there is no feeling after wearing it for seven months.”*

(A9)

### 3.3. Theme 3: Passive Patient Participation during the Treatment Process

The adolescents reported a lack of knowledge and information about the treatment. When the healthcare professionals informed them regarding the brace treatment during the initial consultation, the adolescents felt very helpless and hopeless as the information provided by the healthcare professionals was not sufficient or detailed enough for them to fully comprehend their treatment. They acknowledged that compliance with brace treatment was essential to reduce or prevent the progression of spinal curvature and tried their best to comply with the treatment. Still, they were very passive throughout the treatment process and felt pressured into the treatment plan decided by the healthcare professionals.

*“I didn’t know about the illness much. Then, I was told that bracing was the next step…and I was very emotional at that time.”*

(A1)

*“The prosthetist put the brace on the table and informed me that I needed to wear it, without detailed elaboration of bracing. I felt helpless at that time.”*

(A3)

*“I feel hopeless after knowing my situation. I don’t want to wear the brace.”*

(A6)

Though the adolescents realized that the treatment choice was limited, they hoped the healthcare professionals could address their feeling and difficulties towards bracing. Otherwise, they felt frustrated and helpless whenever they attended medical follow-up consultations, especially when the healthcare professionals could not find a solution for the difficulties encountered.

*“The abrasion between the material and my underarm causes me a lot of pain and bleeding. I have to put tissue paper between my body and the brace to reduce the pain, but it doesn’t work. I told my doctor about that, but he hasn’t figured out a solution to help out. I feel so frustrated and sad. I usually take it off when this happens.”*

(A13)

Since the visual appearance of the brace has negatively affected the adolescents’ feelings and subjective experiences during bracing, they expressed a strong desire to be involved in the visual design of a customized brace if the opportunity would be offered, which could reduce their psychological discomfort regarding the visual appearance of the brace. The customization process could also strengthen their active participation during the treatment process and enhance their motivation to comply with treatment.

*“It will definitely make me feel better if the brace color can be customized, say, match with my skin complexion.”*

(A2)

*“If I can involve in the visual design (e.g., graphic printing) of the brace, it can enhance my willpower in the bracing process regardless of the physical pain…. If I have the chance to participate in the brace design, I won’t feel so scared as what I experienced in the past.”*

(A11)

## 4. Discussion

This study explored the subjective experiences of adolescents with scoliosis and highlighted the obstacles encountered by adolescents during brace treatment. Many of them were not psychologically prepared to be braced at the beginning of the treatment. They acknowledged having a lack of knowledge and information about the brace treatment, and there were limited treatment choices for them to choose from, so they played a relatively passive role during the treatment process, being the receivers of treatment who had to follow what was provided to/planned for them by healthcare professionals. In fact, the barriers and limits to patient participation that we found are consistent with the findings of previous studies conducted in adolescent mental healthcare settings in which participation is restricted to adherence to treatment [21,22]. To enhance the quality of service and achieve better treatment outcomes, there has been an ongoing shift in the direction of person-centered care and shared decision-making in adolescent mental healthcare [23,24,25]. This orientation of healthcare practice has also been expanded to and employed in different healthcare contexts, such as primary care services for acute coronary syndrome and irritable bowel syndrome and outpatient clinics for hypertension [26], though its implementation in clinical contexts poses challenges [27].

Patient participation refers to a process by which patients cooperate in the planning of care or treatment, receive sufficient knowledge about their health and treatment plan, and have a say in their care or treatment [16]. Adolescents’ preferences, values, circumstances, and goals can be incorporated into the treatment process. With the information on evidence-based treatment options provided by health professionals, adolescents and health professionals arrive at a consensus on preferred or optimal person-centered care or treatment through shared decision-making [28]. As a result, the adolescents are empowered in the decisions and actions affecting their lives. Therefore, actively involving adolescents during brace treatment can allow the adolescents to change from a passive role as treatment receivers to active participants. If their obstacles can be recognized and addressed by the healthcare professional, the adolescents feel that they are being understood, respected, and accepted, and it can also lessen their physical discomfort and psychological distress towards bracing, as well as facilitate their motivation to comply with brace treatment. One of the treatment processes that adolescents can actively take part in is the co-design of a customized brace [18]. Consistent with previous findings [29], physical discomfort due to materials and design is one of the biggest obstacles to bracing. Different types of hard and soft braces have been introduced to make the brace smaller and more comfortable and to reduce the stress and/or impairment experienced by adolescents under treatment [6,30]. To achieve corrective effects, it may not be possible to change the materials used for the brace or the overall shape of the brace. However, the visual appearance of the brace as perceived by the adolescents was so scary that their first impression regarding the brace was a feeling of distress. If the adolescents’ feedback on the visual appearance of the brace can be taken into consideration during brace production, by incorporating elements such as adjusting the color of the brace to match the clothes that they frequently wear (e.g., their school uniform) or embedding customized design patterns chosen by the adolescents into the brace, their reluctance caused by the brace’s visual appearance can be reduced, facilitating a more acceptable bracing experience. In addition, the adolescents feel recognized when their opinions and preferences are considered during brace production, which enables the development of a sense of belonging and familiarity with the brace [18].

Apart from physical discomfort due to the materials and design, and reluctance caused by the visual appearance of the brace, concerns regarding their self-image and body configuration during bracing further magnified the psychological distress throughout the treatment process, negatively affecting patients’ brace compliance. One way to deal with psychological distress involves using an appropriate psychosocial intervention, especially in a group setting. It allows adolescents to share their concerns with their peers, helps them seek peer support, and alleviates their distress regarding bracing [15]. Recent systematic reviews and meta-analyses of psychosocial intervention for children and adolescents with other chronic medical conditions have suggested that such interventions can improve outcomes in children and adolescents [31]. Furthermore, psychosocial interventions in group settings have resulted in more positive perceptions of teenagers with similar medical conditions [32] and better adolescent treatment adherence compared with control groups who receive treatment as usual (TAU) [31,33,34,35]. Therefore, psychosocial intervention, including psychoeducation, cognitive restructuring of false beliefs and common misconceptions about braces, strategies for managing stress, coping skills, and aesthetic restructuring through co-designing and personal styling can enhance adolescents’ self-image, reduce psychological distress during bracing, and facilitate positive attitudes and perceptions towards braces, thus ultimately improving the psychosocial well-being of adolescents with scoliosis.

Finally, adolescents reported wearing additional outerwear or loose-fit clothing styles to cover up their braced bodies as no proper styling advice was received from either healthcare professionals or their parents. If adolescents understand how to apply clothing and styling coordination skills to conceal their braced bodies, they can maintain a positive body image and minimize the unpleasant feelings caused by the brace. In addition, adolescents in the developmental stage of puberty are highly concerned with peer recognition and acceptance. Conversations with friends about appearance and criticism of appearances from peers lead to body dissatisfaction [36]. Since individuals’ body shapes, spine deformities, and styling preferences are different, the provision of personalized appearance style management, such as clothing styling, can be integrated into the treatment process as it helps adolescents develop a positive and confident body image via appropriate clothing selection and styling during brace treatment.

In our study, the adolescents were mainly recruited from one of two referral centers in the territory-wide scoliosis screening program, which is a specialized center for the treatment of pediatric orthopedic and spine deformity problems for children/adolescents. Therefore, their subjective experiences during the treatment process may be affected by the organizational routine and rules within that healthcare context, limiting patients’ participation and involvement. In addition, it is conceivable that adolescents’ acceptance factors and reactions to the different aspects of brace treatment may differ between male and female adolescents, resulting in biases in their subjective experiences. Finally, the duration of bracing ranged from 4 to 36 months. Half of them had a duration of fewer than 8 months, while the remaining adolescents had a period of 12 months or more. Though a broad spectrum in the duration of bracing can elicit diverse views and comments on bracing treatment, their subjective experiences and obstacles for brace compliance may change over time. To further understand their subjective experiences throughout the whole treatment process, it is recommended that adolescents who have completed the brace treatment be recruited in the future to explore how they overcame these obstacles.

## 5. Conclusions

This study provided insights into how adolescents with scoliosis experienced brace treatment and what they perceived as obstacles to brace compliance. Active patient participation throughout the treatment process, involving the co-design of customized braces, psychosocial intervention, and personalized appearance style management should be considered and promoted to enhance the patient experience during brace treatment, which will ultimately help adolescents with scoliosis achieve better brace compliance.

## Figures and Tables

**Table 1:** Demographic and clinical information of 15 adolescents with scoliosis.

Participant	Sex	Age at Interview (Years)	Education	School Type	Cobb Angle	Duration of Bracing (Months)
1	Female	10	Primary school	Co-ed	33°	12
2	Female	12	Secondary school	Co-ed	34°	24
3	Female	12	Secondary school	Co-ed	27°	12
4	Female	10	Primary school	Co-ed	35°	4
5	Male	15	Secondary school	Co-ed	35°	5
6	Female	13	Secondary school	All-girls	34°	6
7	Female	14	Secondary school	All-girls	39°	6
8	Female	12	Primary school	Co-ed	33°	7
9	Male	14	Secondary school	Co-ed	37°	7
10	Female	13	Secondary school	Co-ed	33°	15
11	Female	13	Secondary school	All-girls	25°	6
12	Female	13	Secondary school	Co-ed	48°	12
13	Female	14	Secondary school	All-girls	32°	36
14	Female	14	Secondary school	All-girls	30°	24
15	Female	16	Secondary school	Co-ed	34°	24

Co-ed: co-educational.

## Data Availability

The data presented in this study are available upon reasonable request from the corresponding author. The data are not publicly available as they contain information that could compromise the privacy of the adolescents who participated in the study.
